# *Echinacea purpurea* Ethanol Extract Improves Male Reproductive Dysfunction With Streptozotocin–Nicotinamide-Induced Diabetic Rats

**DOI:** 10.3389/fvets.2021.651286

**Published:** 2021-04-28

**Authors:** Chien-Feng Mao, Sabri Sudirman, Chi-Chih Lee, David Tsou, Zwe-Ling Kong

**Affiliations:** ^1^Department of Food Science, National Taiwan Ocean University, Keelung, Taiwan; ^2^Fisheries Product Technology, Faculty of Agriculture, Universitas Sriwijaya, Palembang, Indonesia

**Keywords:** diabetes, *Echinacea purpurea*, inflammation, male reproduction, oxidative stress, Toll-like receptor

## Abstract

As lifestyle changes, the prevalence of diabetes increases every year. Diabetes-induced male reproductive dysfunction is predominantly due to increased oxidative stress and then results in sperm damage and infertility. *Echinacea purpurea* is a traditional medicinal herb and is well-known for its immune-modulatory, antioxidative, anti-inflammatory, anticancer, and antiviral activities. The Toll-like receptor 4 (TLR4) plays a critical role in innate immune responses leading to nuclear factor (NF)-κB phosphorylation and release of proinflammatory cytokines including nitric oxide (NO), interleukin (IL)-1β, and tumor necrosis factor (TNF)-α. However, the relation between *Echinacea purpurea* extract and TLR4 remains unclear. This study aimed to investigate the protective effects on male reproduction of *Echinacea purpurea* ethanol extract (EPE) against diabetic rats and whether the anti-inflammatory effects were through the TLR4 pathway. Diabetic male Sprague–Dawley (SD) rats were induced by streptozotocin (65 mg/kg) and nicotinamide (230 mg/kg). EPE was tested in three doses (93, 279, and 465 mg/kg p.o. daily) for 4 weeks. Besides, metformin administration (100 mg/kg/day) was treated as a positive control. Results indicated that EPE administration for about 4 weeks improved hyperglycemia and insulin resistance. Additionally, EPE increased sperm motility, protected sperm morphology and mitochondrial membrane potential, as well as protein for testosterone synthesis enzyme. In sperm superoxide dismutase, catalase, and glutathione antioxidants were increased, whereas proinflammatory cytokines, such as NO, IL-1β, and TNF-α were decreased. The testis protein content of TLR4 and downstream phospho-NF-κB p65 also were reduced. The EPE might reduce the production of proinflammatory cytokines *via* TLR4 pathways and improve diabetes-induced male infertility.

## Introduction

Diabetes mellitus (DM) has been identified as a metabolic disorder disease. This disease can occur due to insufficient insulin secretion, abnormal insulin action, or both. Type-1 and type-2 DM are the common types of diabetes disease. Type-1 DM is characterized by autoimmune-mediated pancreatic β-cell results in the deficiency of insulin, whereas type-2 DM is peripheral insulin resistance (1). Hyperglycemia was observed in diabetes disease. This condition causes elevated oxidative stress and some proinflammatory cytokine levels, such as interleukin-1β and tumor necrosis factor-α (2, 3). Diabetes disease also causes an adverse effect on organs, such as the liver, pancreas, kidneys, and testis (4). A previous study reported that DM also decreases some steroidogenesis-related genes, such as steroidogenic acute regulatory (StAR) protein, cytochrome P450 enzyme (CYP11A1), and 17β-hydroxysteroid dehydrogenase (HSD) and resulting in impairment of the spermatogenesis and sperm properties (5).

Oral antidiabetic agents have been used for diabetic management. However, some of these agents reported that it increased the prevalence of cardiovascular and gastrointestinal diseases (6). Therefore, the investigation of an alternative antidiabetic agents with less adverse effects is a major topic for future research. Functional foods or natural products are the potential sources for novel antidiabetic agents, such as fucoxanthin from seaweed and antroquinonol-rich extract from *Antrodia cinnamomea* (7, 8).

*Echinacea purpurea* (EP, Asteraceae) is a medicinal plant with an important immunostimulatory effect (9). Extracts of EP have been used in North America for wound and infection treatments (10). This extract also shows antimicrobial and antiviral activities (11). A previous study reported that the bioactive compounds of EP ethanol extract are composed of phenolic acid and isobutylamides. The micro-nanoencapsulated *Echinacea purpurea* ethanol extract has been reported for its ameliorative effects on the diabetic model (12). However, the effect of this ethanol extract alone has been not reported. We hypothesized that EP ethanol extracts alone also have a potential to improve reproductive dysfunction in male diabetic rats. Additionally, the EP ethanol extract shows antioxidant and anti-inflammatory activities (13, 14). Therefore, this study aimed to investigate the ameliorative effects of *Echinacea purpurea* ethanol extract on reproductive dysfunction of streptozotocin–nicotinamide-induced diabetic male rats.

## Materials and Methods

### *Echinacea purpurea* Extraction

*Echinacea purpurea* ethanol extract (EPE) was supplied by the Taiwan Direct Biotechnology Corporation (Taipei, Taiwan). The EPE contains alkylamides (dodecatetraenoic acid isobutylamide) and phenolic compounds (caffeic acid, chlorogenic acid, cichloric acid, and echinacoside) as analyzed by the Taiwan Direct Biotechnology Corporation by using high-performance liquid chromatography (HPLC) assay (12).

### Animals and Treatments

**Figure 1:**
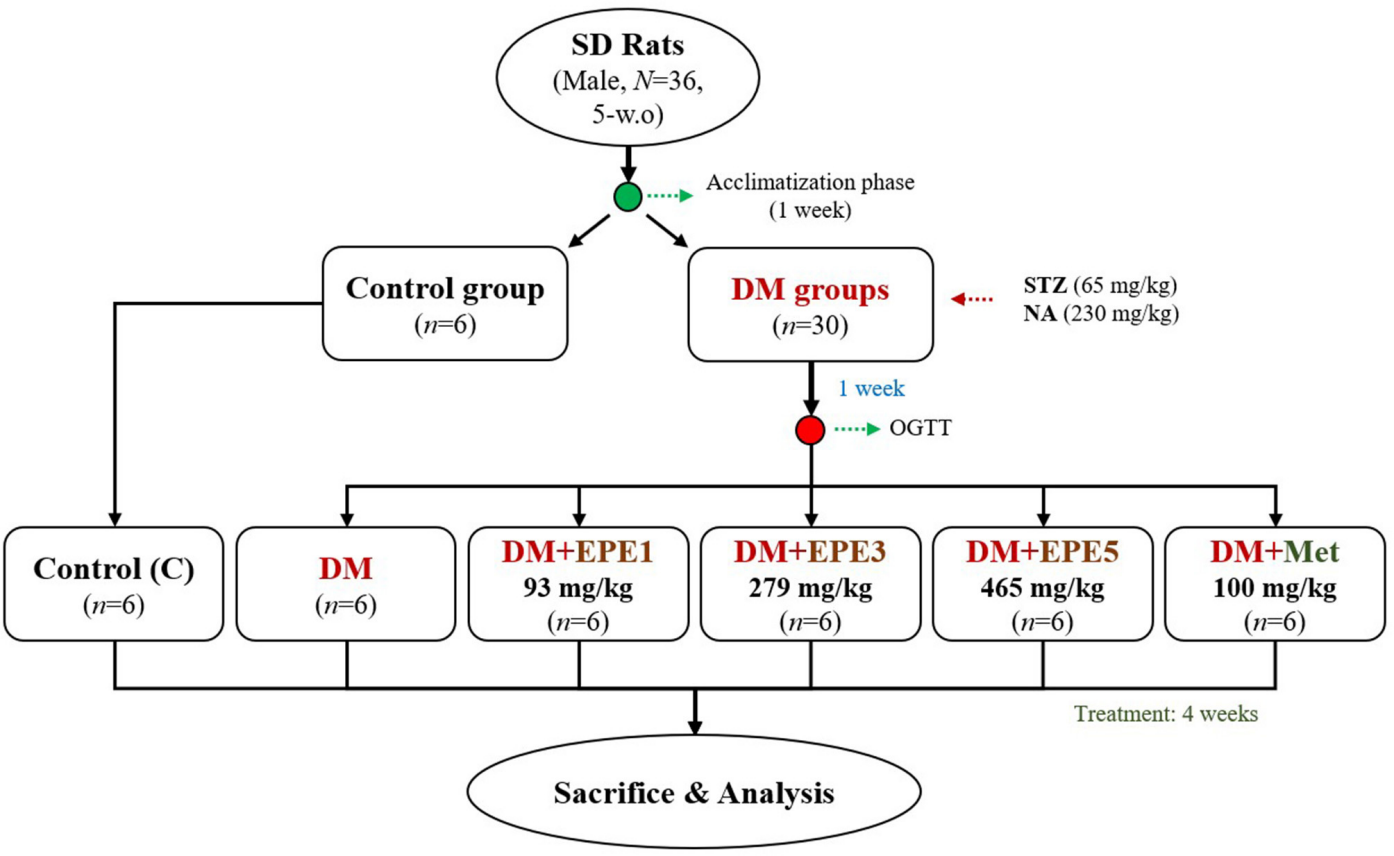
The flowchart of *Echinacea purpurea* ethanol extract treatment against streptozotocin–nicotinamide-induced diabetes male rat model. DM, diabetes mellitus; EPE, *Echinacea purpurea* ethanol extract; Met, metformin.

This study used 36 healthy adult male Sprague–Dawley (SD) rats (*N* = 36, 5 weeks old). The animals were obtained from BioLASCO (Yilan City, Taiwan). They were kept under standard laboratory conditions (12-h light/12-h dark cycle and 23 ± 1°C) and fed a standard rodent diet (LabDiet 5001). Feed and water were provided *ad libitum*. The animal study was reviewed and approved by the Institutional Animal Care and Use Committee (IACUC Approval No. 103033) of the National Taiwan Ocean University. Briefly, the rats were acclimatized for a week and then randomly divided into two main groups (control and diabetes group). The diabetic rat model was intraperitoneally induced by streptozotocin (STZ, 65 mg/kg) and nicotinamide (NA, 230 mg/kg) according to a previous method. The diabetes condition was confirmed by oral glucose tolerance test (OGTT) after a week of STZ–NA injection (15, 16). The rats were confirmed as diabetes if the glucose concentration ≥200 mg/dl at 2-h post-load glucose (17). The diabetes group was divided into five subgroups (*n* = 6) as shown in Figure 1. The first subgroup of diabetic rats without any treatment (DM) and other diabetic rats were daily orally administrated by three different doses of EPE (93, 279, and 465 mg/kg of body weight) for 4 weeks. The EPE doses were chosen according to the previous study (12). Besides, a group of diabetic rats was treated with metformin (100 mg/kg) as a positive control (18), whereas the Control and untreated diabetes (DM) groups were oral gavage administered by distillated water (dH_2_O). The EPE and metformin were dissolved in dH_2_O to make the concentration.

### Oral Glucose Tolerance Test

The oral glucose tolerance test (OGTT) was performed based on the previous methods (15, 19). The OGTT was measured at a week after STZ–NA injection (before the treatment) and the last week of the treatment. Briefly, the rats were fasted for 10 h before the study. Glucose was orally administered (2 g/kg of BW). Blood samples were collected sequentially from the tail vein before and 30, 60, 90, and 120 min after the glucose injection. In OGTT, the glucose level and area under the curve (AUC) were calculated.

### Sample Collection

The rats were sacrificed after treatment for 4 weeks. Whole blood was collected into tubes and centrifuged at 3,000 rpm at 4°C for 15 min to collect the plasma according to the previous method (20). The plasma, testis, and hypothalamus were stored at −80°C until biochemical analysis. Another testis, epididymis, and fat were removed, cleared of adhering connective tissue, and assayed immediately, whereas, sperms in the epididymis were collected by the swim-up technique (21). Briefly, the semen was diluted with two volumes of Roswell Park Memorial Institute (RPMI) 1640 (Gibco, Life Technologies, Grand Island, New York, USA) and then centrifuged at 200 × *g* for 5 min. The supernatant was transferred to another tube. The tube was slanted and incubated for 30 min at 37°C in a 5% CO_2_ incubator for further analysis.

### Plasma Biochemical Assays

The plasma glucose concentration was determined by glucose enzymatic kit (Randox, Colorato, USA). Plasma insulin concentration was measured using a rat insulin enzyme-linked immunosorbent assay (ELISA) kit (Mercodia AB Inc., Sylveniusgatan 8A, Uppsala, Sweden). A homeostasis model assessment of insulin resistance (HOMA-IR) was calculated as fasting plasma insulin concentration (mU/ml) times fasting blood glucose (mmol/L) divided by 22.5 according to a previous study (22). Plasma and testis homogenate IL-1β and TNF-α concentrations were measured using ELISA kits (Peprotech, New Jersey, USA) and rat TNF-α ELISA kits (eBioscience, California, USA) according to the manufacturer's instructions, respectively. The protein concentration in the tissue lysate was determined by the Bradford protein assay (23).

### Antioxidative Analysis and Reactive Oxygen Species Production

The superoxide dismutase (SOD) and catalase activities, as well as reduced glutathione (GSH) level, were observed in sperms of diabetic rats after 4 weeks of treatments. The SOD was determined by the Ransod kit (Randox, Colorato, USA). Catalase activity was measured according to the previously described method (24). The underlying principle of this approach is that the oxygen bubbles generated from the decomposition of hydrogen peroxide (H_2_O_2_) by catalase are trapped by the surfactant Triton X-100. The trapped oxygen bubbles are then visualized as foam, the test-tube height of which is measured to quantify the catalase activity. Briefly, each sample (100 μl) was added in a tube. Subsequently, 100 μl of 1% Triton X-100 and 100 μl of undiluted 30% H_2_O_2_ were added to the solutions and mixed thoroughly and were then incubated at room temperature. Following completion of the reaction, the height of O_2_-forming foam that remained constant for 15 min in the test tube was finally measured using a ruler.

The reduced glutathione (GSH) was estimated by using Ellman's reagent (25). The principle of this approach is that Ellman's reagent (5,5′-dithiobis-2-nitrobenzoic acid) reacts with GSH resulting in a product that can be measured at 412 nm. Briefly, plasma or testis homogenate (500 μl) was mixed with 500 μl of 10% trichloroacetic acid. The contents were mixed well for complete precipitation of proteins and centrifuged at 20,000 × *g* for 5 min. An aliquot of clear supernatant (10 μl) was taken and mixed with 85 μl of PBS. Ellman's reagent (5 μl) was added. After 5 min, the optical density was measured at 412 nm against blank.

The plasma and sperm lipid peroxidation levels were measured according to the concentration of thiobarbituric acid reactive species (TBARs), and the amount of produced malondialdehyde (MDA) was used as an index of lipid peroxidation. The testes were homogenized with buffer containing 1.5% potassium chloride to obtain 1:10 (w/v) whole homogenate. Briefly, one volume of the test sample and two volumes of stock reagent (15%, w/v trichloroacetic acid in 0.25 N HCl and 0.375%, w/v thiobarbituric acid in 0.25 N HCl) were mixed in a centrifuge tube. The solution was heated in boiling water for 15 min. After cooling, the precipitate was removed by centrifugation at 1,500 × *g* for 10 min, and then fluorescence of the supernatant was read at 532 nm against a blank containing all reagents except test sample on a fluorescence spectrophotometer (HITACHI F2000, Tokyo, Japan) (26).

A modified colorimetric nitro blue tetrazolium (NBT) test was used to evaluate superoxide anion production of sperms (27). Briefly, sperm samples, then duplicate samples of 100 μl of washed sperms, were incubated with an equal volume of NBT working reagent (1:10 diluted by RPMI 1640 from 0.01% NBT stock, Sigma-Aldrich, Missouri, USA) at 37°C for 45 min. Following incubation, the samples were washed and centrifuged at 500 × *g* for 10 min in PBS twice to remove all residual NBT solution, leaving only a cell pellet containing formazan. To quantify the formazan product, the intracellular formazan was solubilized in 60 μl of 2 M KOH and dimethyl sulfoxide (DMSO) (Sigma-Aldrich, Missouri, USA), and the resulting color reaction was measured spectrophotometrically on a microplate reader (Dynatech MR5000, Switzerland) at 570 nm.

The nitric oxide in the plasma and testis homogenate of the rats was measured by Griess reagent according to the previous method (28). Briefly, the plasma or sperm homogenate was added to a 96-well plate. Then, 100 μl of Griess solution [sulfanilamide in 5% phosphoric acid and N-(1-naphthyl) ethylenediamine in water mixed in the volume ratio 1:1 immediately before use] was added to the wells and incubated for 10 min. After the incubation time, the absorbance was measured at 570 nm by using a spectrophotometer.

### Epididymal Sperm Concentration, Motility, and Morphology

After the sperm collection, ~10 μl of the diluted sperm suspension was transferred to each counting chamber for counting under a light microscope at 200 × magnification. Sperm progressive motility was evaluated by an earlier method (29). Briefly, the fluid obtained from the cauda epididymis with a pipette was diluted to 2 ml with buffer solution. A slide was placed on a phase-contrast microscope, and an aliquot of this solution was placed on the slide, and percent motility was evaluated visually at 200 × magnification. The method was used for determination of the percentage of morphologically abnormal spermatozoa after adapting the method. A total of 300 sperm cells was examined on each slide, and the head, tail, and total abnormality rates of spermatozoa were expressed as a percentage (30).

### Assessment of Sperm Mitochondrial Membrane Potential

The sperm mitochondrial membrane potential (MMP) was measured by using the fluorescent cationic dye Rhodamine 123 (Sigma-Aldrich, Missouri, USA) according to the previous method (31). Rhodamine 123 dissolved in 0.01 M PBS was added to sperm samples at a final concentration of 10 μM and incubated at 37°C for 30 min. After incubation, the tubes were centrifuged at 800 × *g* for 10 min, and sperms were washed twice and resuspended in PBS. The dye fluorescence to reflect the MMP of sperms was measured using FACS Calibur (Becton Dickinson, San Jose, California, USA).

### Western Blot

Testis and hypothalamus were lysed by using a radioimmunoprecipitation (RIPA) buffer. Hypothalamus tissue was used to measure the G-protein-coupled receptor 54 (GPR54) expression. The protein concentration in the lysates was determined by Bradford protein assay (23). Equal amounts of protein (50 μg) were separated on 10% sodium dodecyl sulfate polyacrylamide gel electrophoresis (SDS-PAGE) gel and, subsequently, electro-transferred onto polyvinyl difluoride (PVDF) membranes. After blocking with 5% skim milk in TBST for 1 h, the membranes were incubated with the primary antibodies at room temperature for 2–3 h or overnight for 4°C. After washing four times with TBST, the membranes were incubated with the appropriated peroxidase-conjugated secondary antibodies at room temperature for 1 h. The antibody dilution is shown in Table 1.

**Table 1:** The antibody dilution.

**Antibody dilution**	**Primary antibody**	**Secondary antibody**
Kiss receptor/GPR54	1:1,000 (rabbit)	1:5,000 (goat anti-rabbit)
StAR	1:1,000 (mouse)	1:5,000 (goat anti-mouse)
CYP11A1	1:1,000 (rabbit)	1:5,000 (goat anti-rabbit)
17β-HSD	1:500 (rabbit)	1:5,000 (goat anti-rabbit)
TLR4	1:1,000 (mouse)	1:5,000 (goat anti-mouse)
phosphor-NF-κB p65	1:500 (rabbit)	1:5,000 (goat anti-rabbit)
α-tubulin	1:5,000 (rabbit)	1:5,000 (goat anti-rabbit)
β-actin	1:5,000 (rabbit)	1:5,000 (goat anti-rabbit)
GAPDH	1:5,000 (rabbit)	1:5,000 (goat anti-rabbit)

### Statistical Analysis

All values were given as mean ± standard error of the mean (SEM). All statistical calculations were done by the SPSS statistics v22.0 (SPSS for Windows Inc., version 22; Chicago, IL, USA) system. One-way ANOVA was used to examine the overall differences between groups, and a Duncan's multiple range test was used to identify significant differences (*p* < 0.05) between the groups.

## Results

### Glucose, Insulin, and Homeostasis Model Assessment of Insulin Resistance Levels

**Figure 2:**
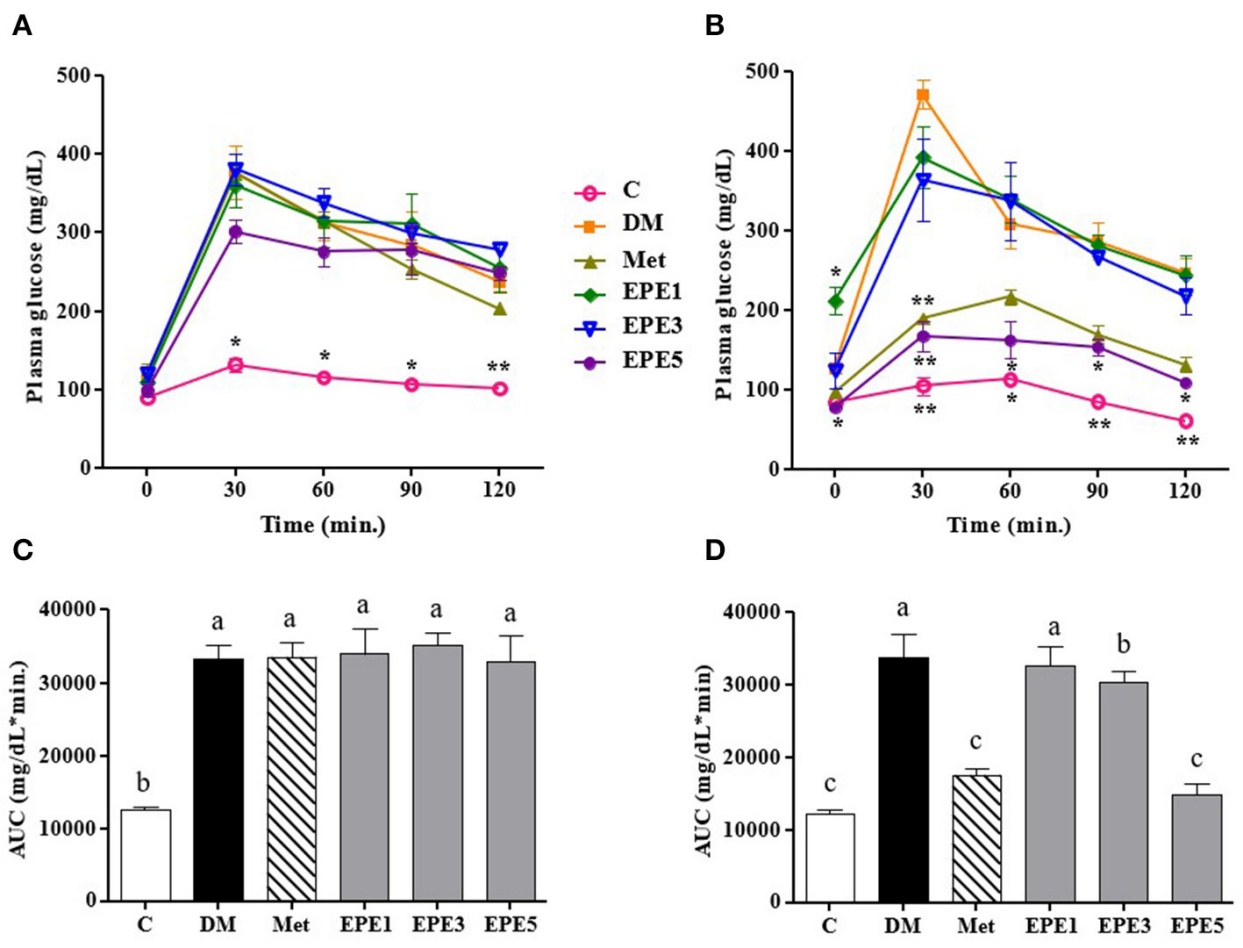
The oral glucose tolerance test (OGTT): a week after streptozotocin (STZ)–nicotinamide (NA) injection or before treatment **(A)** and after treatment **(B)** and the area under the curve (AUC): before treatment **(C)** and after treatment **(D)** in diabetic rats. Data are shown as the mean ± SEM (*n* = 6). Significant difference at *p < 0.05 and **p < 0.01 vs. DM, respectively. The values with different superscript letters (a–c) represent significant differences (*p* < 0.05) as analyzed by Duncan's multiple range test. C, control; DM, diabetes mellitus; Met, metformin; EPE, *Echinacea purpurea* ethanol extract.

Figure 2A showed that the glucose level of high dose of EPE (EPE5) and Met groups significantly decreased after 4 weeks of treatment when compared with the untreated diabetes (DM) group. The glucose of medium dose (EPE3) also significantly decreased after 4 weeks of treatment as shown in Figure 2B and Table 2 when compared with the DM group, whereas there are no significant effects on the insulin level. However, the homeostasis model assessment of insulin resistance (HOMA-IR) level increased in the DM group, and its level significantly reduced in the Met, EPE3, and EPE5 groups after treatment for 4 weeks when compared with the DM group.

**Table 2:** Plasma fasting blood glucose level, plasma insulin level, and homeostasis model assessment of insulin resistance (HOMA-IR) in diabetic rats after 4 weeks of treatments.

**Properties**	**C**	**DM**	**Met**	**EPE1**	**EPE3**	**EPE5**
Glucose (mg/dl)	92.83 ± 16.62^b^	162.96 ± 17.17^a^	104.63 ± 14.86^ab^	119.41 ± 20.09^ab^	80.33 ± 19.61^b^	92.59 ± 20.68^b^
Insulin (μU/ml)	4.67 ± 1.47^a^	5.74 ± 1.60^a^	3.62 ± 0.40^a^	5.36 ± 1.90^a^	5.13 ± 1.93^a^	4.70 ± 1.40^a^
HOMA-IR	1.35 ± 0.13^b^	4.59 ± 0.45^a^	1.32 ± 0.09^b^	4.33 ± 1.28^a^	2.23 ± 0.41^b^	1.43 ± 0.33^b^

*Data are shown as the mean ± SEM (n = 6). The values with different superscript letters (a, b) in the same row represent significant differences (p < 0.05) as analyzed by Duncan's multiple range test. HOMA-IR = fasting plasma glucose (mmol/L) × fasting plasma insulin (μU/ml)/22.5. C, control; DM, diabetes mellitus; Met, metformin; EPE, Echinacea purpurea ethanol extract*.

### Antioxidative Properties and Reactive Oxygen Species Levels

**Figure 3:**
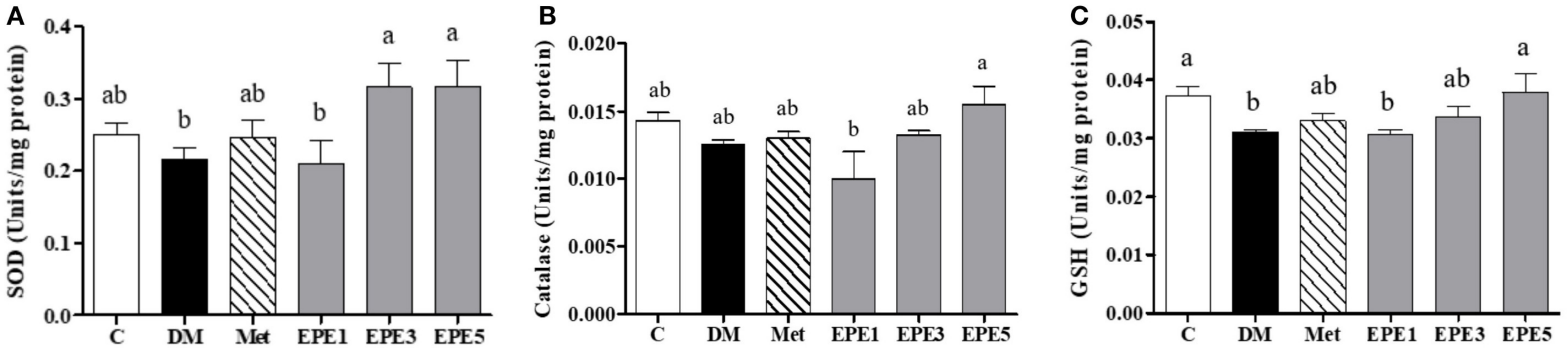
The activities of **(A)** superoxide dismutase (SOD), **(B)** catalase, and **(C)** reduced type glutathione (GSH) of diabetic rats' sperm after 4 weeks of treatments. Data are shown as the mean ± SEM (*n* = 6). The values with different superscript letters (a, b) represent significant differences (*p* < 0.05) as analyzed by Duncan's multiple range test. C, control; DM, diabetes mellitus; Met, metformin; EPE, *Echinacea purpurea* ethanol extract.

The activity of enzymatic antioxidants of the sperm were evaluated after 4 weeks of treatments, such as superoxide dismutase (SOD), catalase, and reduced-type glutathione (GSH) (Figure 3). Low SOD activity was shown in the DM group (Figure 3A). However, after treatment with the medium and high doses of EPE, the SOD activities were significantly enhanced. The catalase activity was observed to increase in the EPE5 group; however, there was no significant effect (Figure 3B), whereas the reduced type of GSH significantly increased in the EPE5 group when compared with the DM group as shown in Figure 3C.

**Figure 4:**
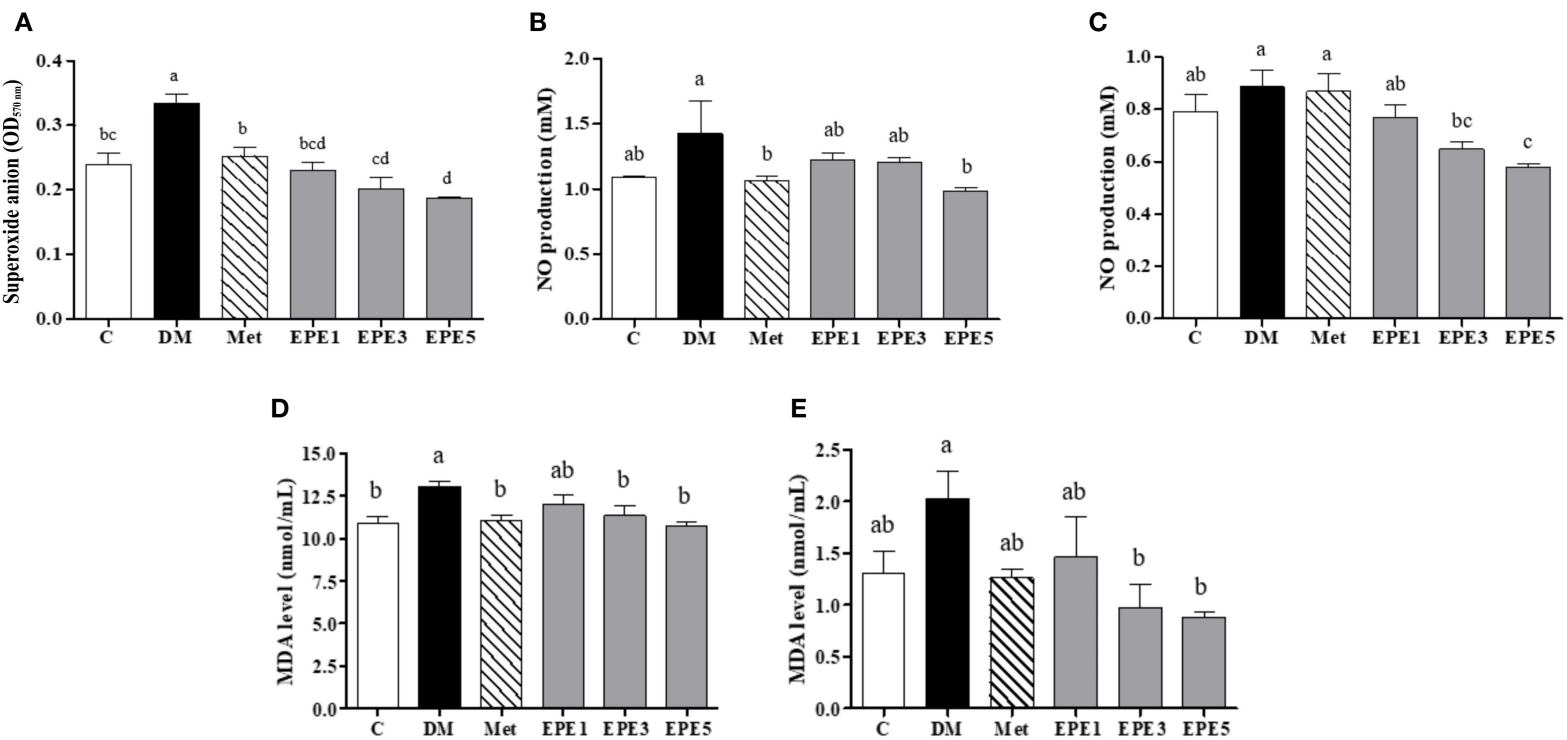
The levels of **(A)** sperm superoxide anion, **(B)** plasma nitric oxide (NO), **(C)** sperm NO, **(D)** plasma malondialdehyde (MDA), and **(E)** sperm MDA in diabetic rats after 4 weeks of treatment. Data are shown as the mean ± SEM (*n* = 6). The values with different superscript letters (a–d) represent significant differences (*p* < 0.05) as analyzed by Duncan's multiple range test. C, control; DM, diabetes mellitus; Met, metformin; EPE, *Echinacea purpurea* ethanol extract.

The high levels of superoxide anion (${\text{O}}_{2}^{-}$), nitric oxide (NO), and malondialdehyde (MDA) in sperm and plasma were observed in the DM group (Figure 4). The levels of superoxide anion, NO, and MDA significantly reduced in sperms after treatment with medium and high doses of EPE (EPE3 and EPE5) as shown in Figures 4A,C,E. The NO and MDA productions in plasma also significantly decreased after treatment with EPE3 and EPE5 (Figures 4B,D). As a positive control, metformin administration also showed significant effects on productions of superoxide anion in the sperm and NO and MDA in the plasma.

### Toll-Like Receptor, Phosphorylated p65 Subunit of NF-κB, and Proinflammatory Cytokines Expressions

**Figure 5:**
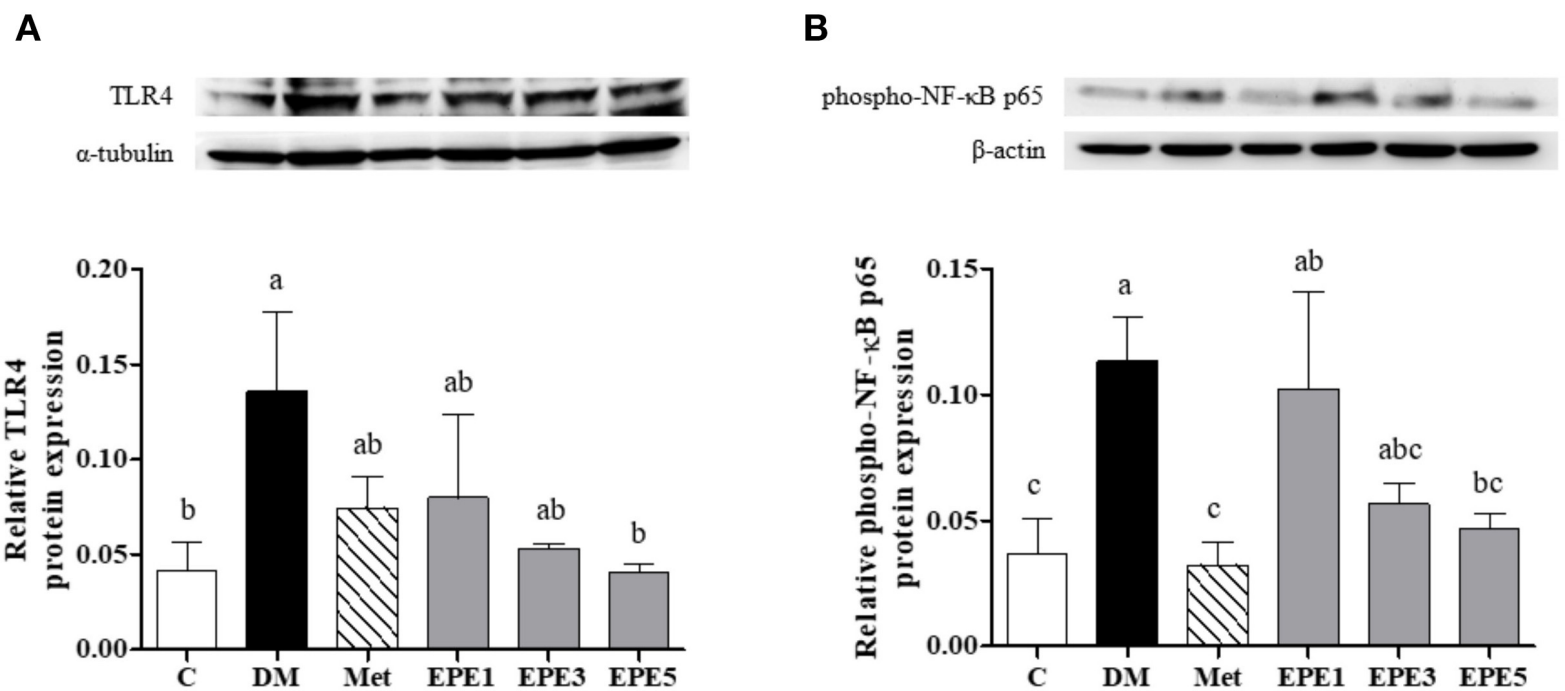
Protein expression of **(A)** Toll-like receptor 4 (TLR4) and **(B)** phosphor-NF-κB p65 in the testes of diabetic rats after 4 weeks of treatments. Data are shown as the mean ± SEM (*n* = 6). The values with different superscript letters (a–c) represent significant differences (*p* < 0.05) as analyzed by Duncan's multiple range test. C, control; DM, diabetes mellitus; Met, metformin; EPE, *Echinacea purpurea* ethanol extract.

Figure 5 shows that the relative expressions of Toll-like receptor 4 (TLR4) and phosphorylated p65 subunit of NF-κB (phosphor-NF-κB p65) increased in the DM group. These levels significantly reduced after treatment with a high dose of EPE (EPE5) for 4 weeks when compared with the DM group. Metformin also significantly reduced phosphor-NF-κB p65 expression in the rats' testes.

**Figure 6:**
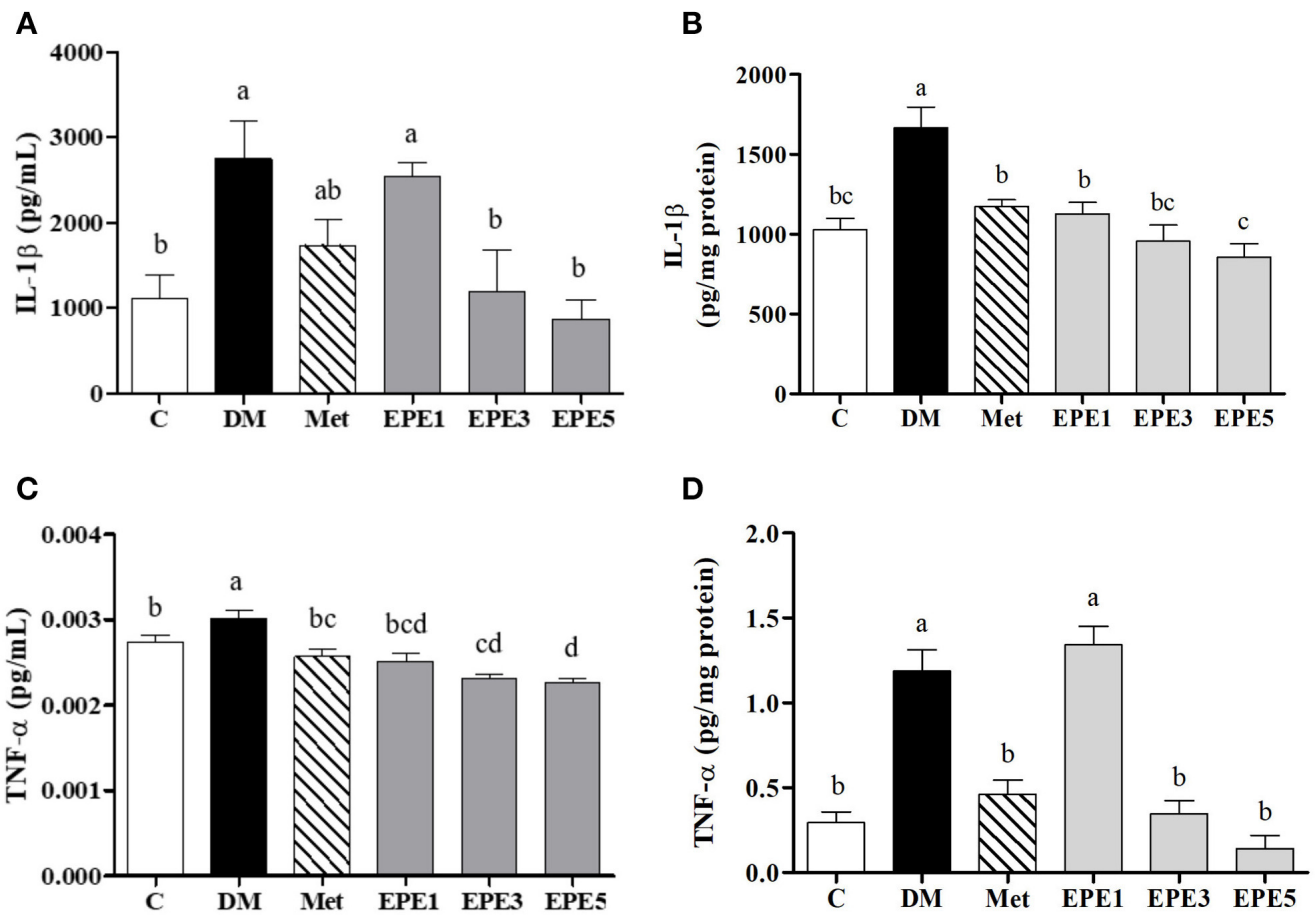
The levels of **(A)** plasma interleukin (IL)-1β, **(B)** testis IL-1β, **(C)** plasma tumor necrosis factor (TNF)-α, and **(D)** testis TNF-α in diabetic rats after 4 weeks of treatments. Data are shown as the mean ± SEM (*n* = 6). The values with different superscript letters (a–d) represent significantly differences (*p* < 0.05) as analyzed by Duncan's multiple range test. C, control; DM, diabetes mellitus; Met, metformin; EPE, *Echinacea purpurea* ethanol extract.

Figure 6 showed that the levels of proinflammatory cytokine increased in the plasma and testes of the DM group, such as interleukin (IL)-1β and tumor necrosis factor (TNF)-α. As shown in Figures 6A–D, these levels were significantly reduced after treatment with medium and high doses of EPE. As a positive control, metformin also significantly reduced the TNF-α level.

### Kiss-1 Peptide Receptor, Testosterone Synthesis Enzymes, and Testosterone Expressions

**Figure 7:**
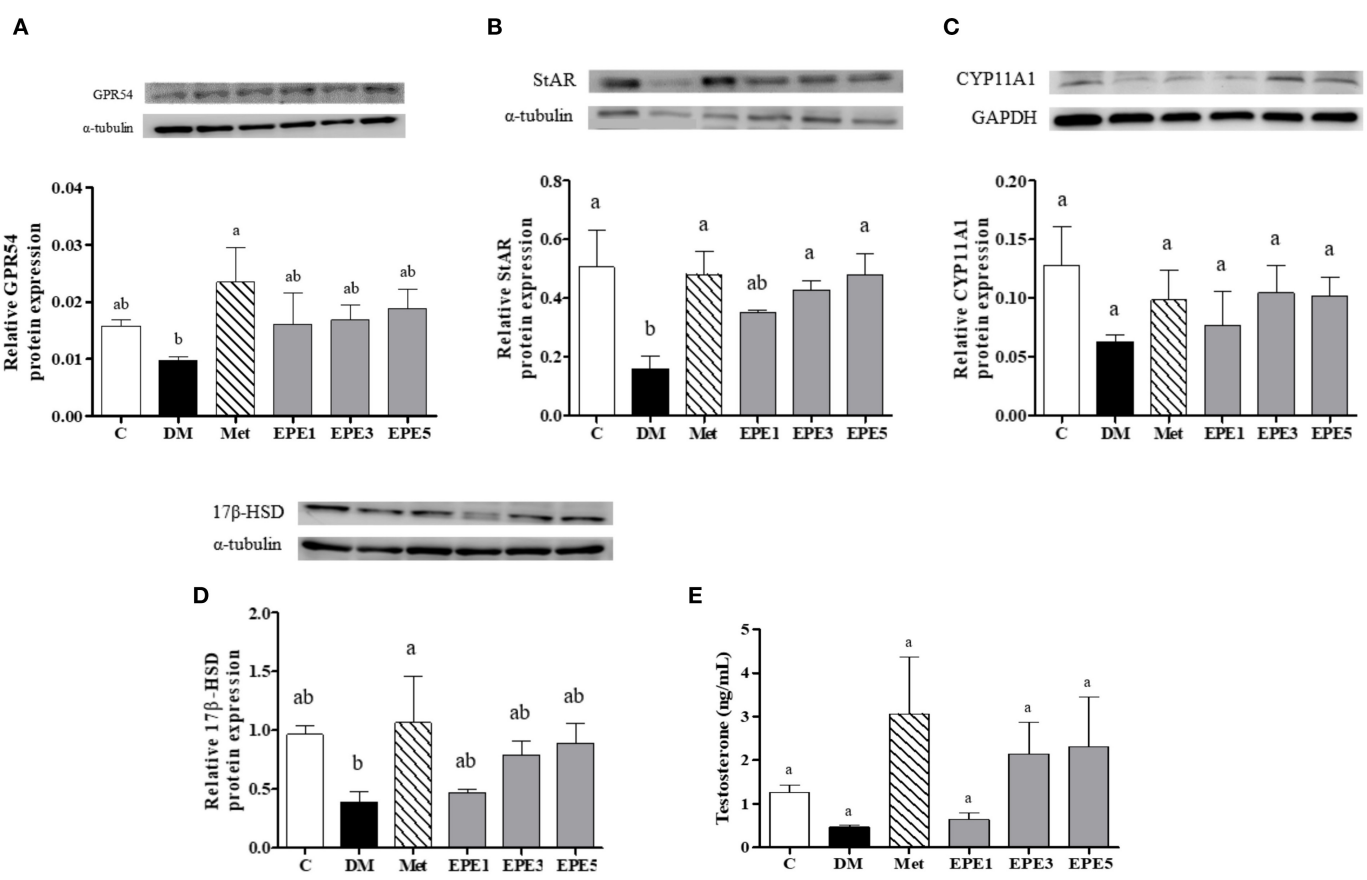
Protein expression of **(A)** GPR54 (Kiss-1 receptor) in hypothalamus, **(B–D)** testosterone synthesis enzymes in testis, and **(E)** plasma testosterone level in diabetic rats after 4 weeks of treatment. Data are shown as the mean ± SEM (*n* = 6). The values with different superscript letters (a,b) represent significant differences (*p* < 0.05) as analyzed by Duncan's multiple range test. C, control; DM, diabetes mellitus; Met, metformin; EPE, *Echinacea purpurea* ethanol extract.

Figure 7A showed that the relative expression of G-protein-coupled receptor (GPR54/Kiss-1 peptide receptor) decreased in the hypothalamus of the DM group. This level increased after treatment with metformin and EPE; however, there are no significant effects for the expression. The expressions of some testosterone synthesis enzymes, such as StAR, CYP11A1, and 17β-HSD proteins also reduced in the testes of the DM group (Figures 7B–D). There are no effects on the relative expression of the CYP11A1 protein after treatment with EPE. The expression of the 17β-HSD protein increased after treatment with EPE for 4 weeks; however, there are no significant effects. The StAR protein expression significantly restored after treatment with metformin, and medium and high doses of EPE (EPE3 and EPE5). Additionally, there are no significant effects on plasma testosterone level as shown in Figure 7E.

### Mitochondria Membrane Potential and Sperm Properties

**Figure 8:**
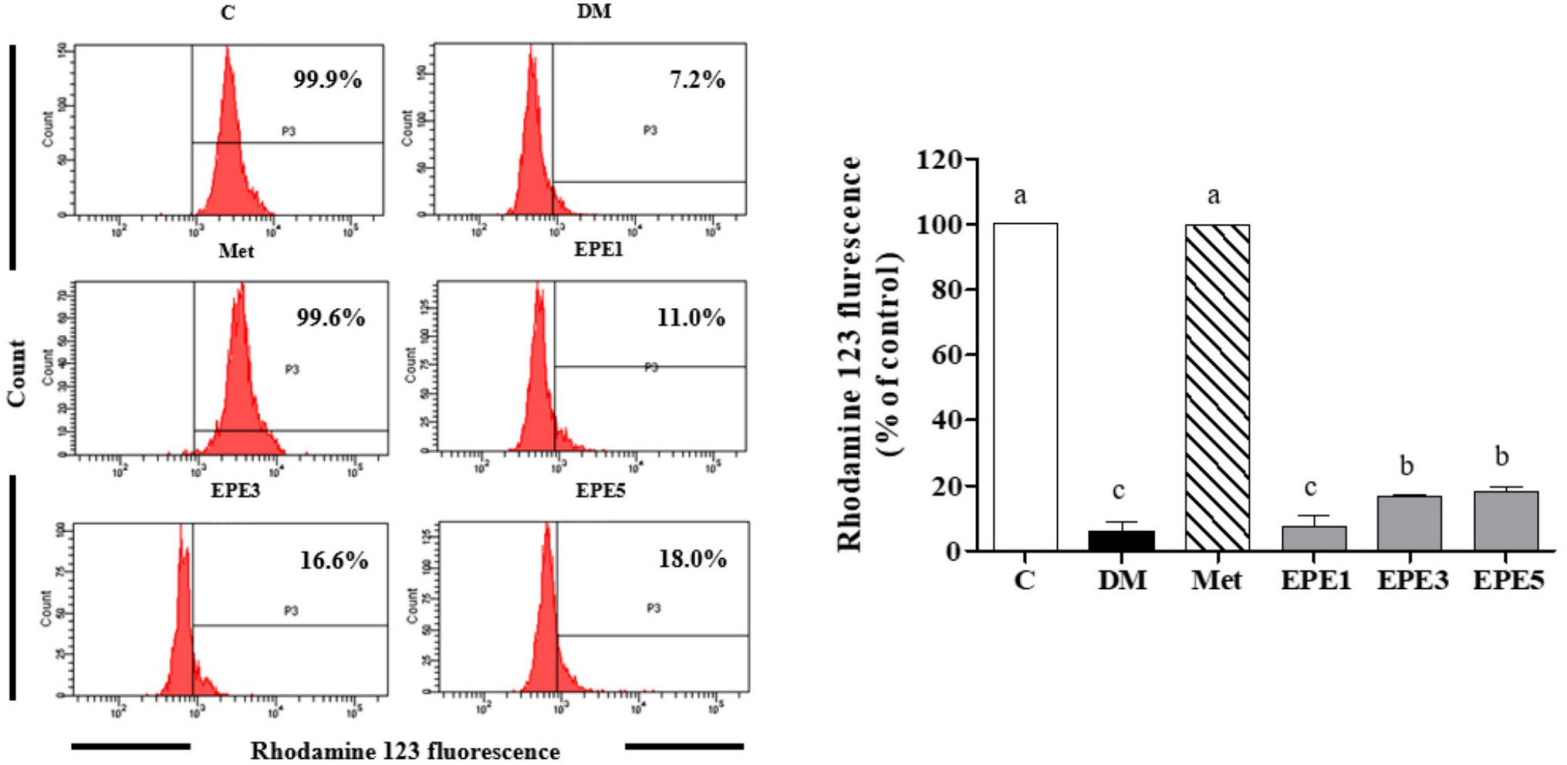
The levels of mitochondria membrane potential (MMP) of sperm in diabetic rats after 4 weeks of treatments. Data are shown as the mean ± SEM (*n* = 6). The values with different superscript letters (a–c) represent significant differences (*p* < 0.05) as analyzed by Duncan's multiple range test. C, control; DM, diabetes mellitus; Met, metformin; EPE, *Echinacea purpurea* ethanol extract.

A low level of mitochondria membrane potential (MMP) was shown in the DM group (Figure 8). EPE and metformin treatment for 4 weeks significantly protected the mitochondrial function by restoring the level of MMP. Table 3 shows that there are no effects on total sperm count after treatment with EPE for 4 weeks. However, EPE treatment significantly increased sperm progressive motility and decreased sperm abnormalities.

**Table 3:** The total sperm count, sperm progressive motility, and sperm abnormalities of diabetic rats after 4 weeks of treatments.

**Sperm properties**	**C**	**DM**	**Met**	**EPE1**	**EPE3**	**EPE5**
Total count (10^5^)	41.70 ± 3.21^a^	38.80 ± 1.28^a^	35.50 ± 4.40^a^	37.60 ± 1.90^a^	38.10 ± 3.08^a^	41.2 ± 7.60^a^
Progressive motility (%)	18.53 ± 1.18^a^	9.03 ± 2.06^c^	16.61 ± 2.06^ab^	12.07 ± 1.70^bc^	16.12 ± 0.62^ab^	20.17 ± 1.71^a^
Abnormalities (%)	2.10 ± 0.63^d^	11.73 ± 0.91^a^	4.74 ± 0.63^c^	9.06 ± 1.11^b^	4.24 ± 0.22^cd^	3.30 ± 0.21^cd^

*Data are shown as the mean ± SEM (n = 6). The values with different superscript letters (a–d) in the same row represent significant differences (p < 0.05) as analyzed by Duncan's multiple range test. C, control; DM, diabetes mellitus; Met, metformin; EPE, Echinacea purpurea ethanol extract*.

## Discussion

In this study, we succeeded in demonstrating the ameliorated effects of *Echinacea purpurea* ethanol extract (EPE) on oxidative stress, proinflammatory cytokines, and sperm properties associated with reproductive dysfunction of diabetic male rats. The diabetic rats were induced intraperitoneally by streptozotocin–nicotinamide (STZ-NA). Then the rats were treated with three doses of EPE and metformin (a positive control) for 4 weeks. Failure of insulin action is a characteristic of type 2 diabetes and also known as non-insulin-dependent diabetes mellitus (NIDDM) (32).

In this present study, the diabetes condition was confirmed by high levels of glucose and the homeostasis model assessment of insulin resistance (HOMA-IR) as shown in Figure 2 and Table 2. A previous reference reported that if the glucose concentration ≥200 mg/dl at 2 h post-load, glucose in the OGTT was confirmed as a provisional diagnosis of diabetes (17). The STZ–NA injection increases glucose levels in the diabetic animal model. This condition, due to STZ injection, triggers damage of the pancreatic β-cells, and the cell was partially protected from STZ by NA (15). An experiment with an STZ–NA to induce a diabetic condition also shows high levels of plasma glucose (2-h OGTT ≥200 mg/dl) and HOMA-IR in rat models as reported by a previous study (7). Additionally, a previous study also reported that 65 mg/kg of STZ with 230 mg/kg of NA injection was confirmed as a diabetes condition (15). The high dose of EPE administration successfully decreased plasma glucose and HOMA-IR levels of diabetic rats after 4 weeks of treatment.

Reducing enzymatic antioxidant activities especially superoxide dismutase (SOD) was observed in the untreated diabetes (DM) group. It also showed an increase in oxidative stress markers such as superoxide anion (${\text{O}}_{2}^{-}$), nitric oxide (NO), and malondialdehyde (MDA) in plasma and sperm as shown in Figures 3, 4. Reactive oxygen species (ROS) including ${\text{O}}_{2}^{-}$ have been implicated in diabetes pathology, and they are involved in cell damage and insulin resistance (33). A high level of superoxide anion causes tissue damage. However, the presence of SOD protects tissues from oxidative damage by converting ${\text{O}}_{2}^{-}$ to hydrogen peroxide (H_2_O_2_) (34). The SOD activity was enhanced by treatment with medium and high doses of EPE. This condition also results in a reduction of oxidative stress markers. Additionally, the reduced type of glutathione (GSH) also increased after high doses of EPE treatment. High levels of GSH confirmed that the EPE extract successfully ameliorates oxidative stress in the diabetic model. A low level of GSH is a marker for oxidative stress conditions (35).

A high level of Toll-like receptor 4 (TLR4) and phosphorylated p65 subunit of NF-κB (phospho-NF-κB p65) was observed in the untreated diabetes (DM) group (Figure 5). Toll-like receptor 4 is a cell surface receptor that involves immune responses by triggering activation of transcription factor and kinase cascade signaling. The TLR4 also involves insulin resistance (IR) and inflammation developments. TLR4 is also an upstream regulator of nuclear factor (NF)-κB activation. A high level of proinflammatory cytokines, such as interleukin (IL)-1β and tumor necrosis factor (TNF)-α, was observed as the cascade signaling the TLR4 receptor (36). This present study also reported a high expression of phosphorylated p65 subunit of NF-κB and a high level of IL-1β and TNF-α (Figure 6). A previous study reported that activation of NF-kB transcription factor plays an important role in diabetes complications. Additionally, NF-kB activation is caused by oxidative stress (37). A high blood glucose or a hyperglycemia condition has been considered to trigger oxidative stress and also increase proinflammatory cytokines, such as IL-1β and TNF-α (38, 39). These protein expressions were successfully increased by EPE administration after 4 weeks of treatment.

An untreated diabetes (DM) group showed a low expression of Kiss1 protein receptor (G-protein-coupled receptor 54, GPR54) (Figure 7). A low expression of this protein might be caused by the increasing level of proinflammatory cytokines. Additionally, a previous study reported that GPR54 expression was reduced by TNF-α (40). This condition improved by EPE treatment. In this study, we also observed some steroidogenesis-related genes, such as steroidogenic acute regulatory (StAR) protein, cytochrome P450 enzyme (CYP11A1), and 17β-hydroxysteroid dehydrogenase (HSD). A low expression of StAR protein was shown in the untreated diabetes (DM) group. The StAR protein plays an important role in the biosynthesis of steroid hormone. It acts as mediator of cholesterol transport across the mitochondrial membrane during steroidogenesis, whereas cholesterol has been known as a common precursor substrate of steroid hormones (41, 42). The StAR protein expression was successfully improved after EPE treatment.

This present study also reported a low level of mitochondrial membrane potential (MMP) in the untreated diabetes (DM) group. This condition was successfully ameliorated by EPE treatment (Figure 8). The MMP has been used to measure the mitochondrial function as an indicator of cell health (43). A previous study reported that male fertility can be affected by diabetes. This condition was characterized by low DNA integrity and sperm motility (44). The EPE treatment successfully increased sperm motility and reduced the sperm abnormalities (Table 3).

## Conclusion

The streptozotocin–nicotinamide injection successfully induced diabetic conditions and is involved with male reproductive dysfunction. Diabetes disease was characterized by a high glucose level (hyperglycemia) and caused oxidative stress as well as regulated Toll-like receptor expression, increased expression of nuclear factor-kappa B transcription factor, and proinflammatory cytokines, whereas male reproductive dysfunction was characterized by low expression of G-protein-coupled receptor, mitochondrial membrane receptor, and low quality of the sperm. However, after 4 weeks of oral administration of *Echinacea purpurea* ethanol extract, diabetes condition was successfully ameliorated and male reproductive dysfunction was also improved.

## Data Availability Statement

The original contributions presented in the study are included in the article/supplementary material, further inquiries can be directed to the corresponding author.

## Ethics Statement

The animal study was reviewed and approved by Institutional Animal Care and Use Committee (IACUC Approval No. 103033) of the National Taiwan Ocean University.

## Author Contributions

Z-LK conceptualized the study. C-CL and C-FM conducted the formal analysis. C-CL and SS wrote the original draft. DT, SS, and Z-LK wrote, reviewed, and edited the article. All authors have read and agreed to the published version of the manuscript.

## Conflict of Interest

The authors declare that the research was conducted in the absence of any commercial or financial relationships that could be construed as a potential conflict of interest.
